# The changes of hepatic bile acid synthesis and transport and bile acids profiles in isopsoralen-induced liver injury C57BL/6J mice

**DOI:** 10.1080/13880209.2022.2116057

**Published:** 2022-09-06

**Authors:** Wei-jie Men, Zhao-jun Meng, Qin Wang, Meng-ying Chen, Yu-xia Zhai, Hong Shi, An-hong Wang, Kun Zhou

**Affiliations:** a Institute of Traditional Chinese Medicine, Tianjin University of Traditional Chinese Medicine, Tianjin, China; b Tianjin Institute of Pharmaceutical Research Co., Ltd, Tianjin, China; c Tianjin Key Laboratory of Chinese Medicine Pharmacology, Tianjin, China; d Department of Pharmacy, Gansu Provincial Hospital, Lanzhou, Gansu, China; e State Key Laboratory of Component-based Chinese Medicine, Tianjin, China

**Keywords:** Target organs, metabolism, hepatotoxicity, BAs transporters, BAs spectrum

## Abstract

**Contest:**

Isopsoralen, one of the main active and quality-control compounds in *Psoralea corylifolia* L. (Fabaceae), has antitumor and oestrogen-like effects. Previous studies demonstrated that isopsoralen induced hepatotoxicity and its long-term exposure led to cholestatic liver injury.

**Objective:**

This study investigates the effect of three- or seven-day exposure of low dose isopsoralen (80 mg/kg) on bile acid homeostasis in C57BL/6J mice.

**Materials and methods:**

Forty-two C57BL/6J mice were randomly divided into control, three- and seven-day groups (*n* = 14 per group, half female and half male). Isopsoralen suspension was administrated intragastrically at 80 mg/kg once a day. Blood and liver samples were collected to measure biochemical indices and transport of BAs. The histopathology of the liver was also observed. HPLC–MS/MS was also used to measure the BAs profiles and transport activity.

**Results:**

In the study, isopsoralen increased the levels of serum AST, ALT in three- and seven-day groups, and caused vacuolar degeneration and swelling in the liver. Canalicular efflux transporters BSEP, OSTα, MRP2, MRP3, and basolateral uptake transporters NTCP, OATP4 were inhibited after seven-day-administration. Moreover, amino acid binding enzymes (BAAT and BACS) were also inhibited after seven-day-administration. The composition of BAs changed greatly and the concentration of some unconjugated-BAs which have stronger hydrophobicity, such as CA, CDCA, was significantly increased.

**Conclusions:**

Isopsoralen (80 mg/kg) caused hepatotoxicity after short-term exposure by inhibiting the expression of efflux transporters, amino acid binding enzymes, and disrupting BAs spectrum.

## Introduction

Fructus Psoraleae (FP) is a commonly used herbal medicine to treat psoriasis, osteoporosis and vitiligo in southeast Asia (Lu 1983; Alam et al. 2018; Koul et al. 2019). Recently, FP was shown to induce liver injury that manifested histologically as cholestasis, necroses, degeneration and inflammatory cell infiltration (Nam et al. 2005; Cheung et al. 2009; Zhang P et al. 2016). Isopsoralen, which is a furocoumarin, is used as a quality control compound of FP as described in Pharmacopoeia of People’s Republic of China. It presents many pharmacological properties, such as anti-osteoporotic effects, antioxidant, antitumor and oestrogen-like action (Latha et al. 2000; Kong et al. 2001; Xiao et al. 2010; Xin et al. 2010; Li XM et al. 2017; Ge et al. 2018). Moreover, isopsoralen is also the main culprit for the hepatotoxicity of FP. Long-term or high dose exposure to isopsoralen leads to histopathology changes in the liver and deranged liver function indicated by elevated serum aspartate transaminase (AST), alanine transaminase (ALT) (Zhang Y, Yuan, et al. 2019; Zhang Y, Zhang, et al. 2019). The obstruction of bile acid (BA) excretion has been regarded as an important cause of liver injury (Attili et al. 1986; Li M et al. 2017), clinical studies have demonstrated that isopsoralen could cause cholestatic liver damage by down-regulation of bile salt export pump (BSEP) and other bile-related transporters (Wang J et al. 2012).

The abnormal expression of BA transporters is usually accompanied by the imbalance of BA homeostasis. Primary BAs, cholic acid (CA), chenodeoxycholic acid (CDCA) are usually conjugated to glycine or taurine in liver by BA-CoA: amino acid N-acyltransferase (BAAT) and BA CoA synthetase (BACS). Then they are secreted into the gut and modified by intestinal flora into secondary BAs, such as deoxycholic acid (DCA) and lithocholic acid (LCA) (Chiang 2013). BAs regulate the composition of intestinal flora and usually exist in the form of membrane-impermeable anions (Stacey and Webb 1947; Kullak-Ublick et al. 2004; Kurdi et al. 2006). To maintain the steady-state balance of BAs in liver, there are efflux transporters, such as BSEP, organic solute transporters alpha (OSTα), multidrug resistance-associated protein 2 (MRP2), multidrug resistance-associated protein 3 (MRP3), multidrug resistance-associated protein 4 (MRP4) and uptake transporters of BAs, such as Na^+^ taurocholic co-transporting polypeptide (NTCP), organic anion transporter polypeptide 2 (OATP2), organic anion transporter polypeptide 4 (OATP4) localized to the apical or the basolateral membrane of polarized epithelial hepatocytes (Trauner and Boyer 2003).

In this study, the liver injury model induced by isopsoralen was established in C57BL/6J mice to investigate changes in serum biochemistry, BA profiles and BA transporters further.

## Materials and methods

### Drugs and reagents

Isopsoralen (purity >98% by HPLC) was purchased from Chengdu Pufeide Biotech Co, Ltd. (Chengdu, China). The vesicles expressing the transporter proteins of human BSEP (GM0005), MRP2 (GM0001) and MRP4 (GM0012) were bought from GenoMembrane Co., Ltd. (Yokohama, Japan). Rifampin (130496), benzbromarone (100677) and indomethacin (100258) were purchased from National Institutes for Food and Drug Control (Beijing, China). Deoxylithocholic acid (DLCA), NCA, allocholic acid (ALCA) and ursocholic acid (UCA) were purchased from Truth and Reconciliation Commission (Winnipeg, Canada); LCA, CA, CDCA, DCA, hyodeoxycholic acid (HDCA), ursodeoxycholic acid (UDCA), taurodeoxycholic acid (TDCA), taurolithocholic acid (TLCA), glycoursodeoxycholic acid (GUDCA), taurochenodeoxycholic acid (TCDCA), taurocholic acid (TCA), glycodeoxycholic acid (GDCA), glycocholic acid (GCA), glycochenodeoxycholic acid (GCDCA), apocholic acid (APCA), 12-dehydrocholic acid (12-DHCA), murocholic acid (MoCA), 3-dehydrocholic acid (3-DHCA), 7-dehydrocholic acid (7-DHCA), 23-norcholic acid (NCA), 6,7-diketolithocholic acid (6,7-DKLCA), 23-nordeoxycholic acid (NDCA), β-muricholic acid (β-MCA) and tauro-β-muricholic acid (T-βMCA) were purchased from Sigma (Sigma, St. Louis, MO).

ALT, AST, alkaline phosphatase (ALP), total BA (TBA), total triglyceride (TG), total bilirubin (TBIL), total protein (TP) and total cholesterol (TC) kits were purchased from BioSino Biotechnology and Science Inc. (Beijing, China). Acetonitrile and methanol were bought from Merck Chemicals (Kenilworth, NJ). Formic acid was purchased from CNW (Düsseldorf, Germany). Phenylmethanesulfonyl fluoride (PMSF) and radio immunoprecipitation assay (RIPA) lysis buffer were purchased from Beijing Solarbio Science & Technology Co., Ltd. (Beijing, China). Primary antibodies to MRP2 (BA1667), NTCP (PB0788), OSTα (A10127) and farnesoid X receptor (FXR) (A00835-1) were purchased from BOSTER Biological Technology Co., Ltd. (Wuhan, China). Antibodies to BSEP (sc-74500), OATP2 (sc-376424) and OATP4 (sc-376904) were purchased from Santa Cruz Biotechnology (Dallas, TX). The primary antibody to MRP4 (ab180712) was purchased from Abcam (Cambridge, MA). The primary antibodies to MRP3 (#39909) and β-actin (#4970) were purchased from Cell Signaling Technology (Danvers, MA). The primary antibody to BAAT was purchased from ABclonal Technology Co., Ltd. (Wuhan, China). Primary antibody to BACS was purchased from SAB (Greenbelt, MD). Anti-mouse IgG HRP-linked antibody (#7076) and anti-rabbit IgG HRP-linked antibody (#7074) were purchased from Cell Signaling Technology (Danvers, MA).

### Animal and treatment

Eight-week-old C57BL/6J mice (weighing 18–22 g) were obtained from Beijing HFK Bioscience Technology Co., Ltd. (Beijing, China) and housed under a 12 h light/dark cycle. Water and food can be accessed freely and all animals were fed for 1 week to acclimatize the laboratory conditions. All procedures were approved by the Laboratory Animal Ethics Committee of Tianjin University of Traditional Chinese Medicine (permit number: TCM-LAEC 2019049, 5 March 2019).

Twenty-two C57BL/6J mice were randomly divided into three groups (*n* = 14 per group, half female and half male), control group (Cont), 3-day (3d) and 7-day (7d). The control group was intragastrically treated for seven days with pure water. In the three-day group, pure water was given once a day for the first four days, then isopsoralen suspension was administrated intragastrically at 80 mg/kg for the next three days. In the seven-day group, 80 mg/kg isopsoralen suspension was administrated intragastrically for seven days. Moreover, the body weights of all animals were measured once a day, and according to the body weight to adjust the volume of isopsoralen or pure water.

### Sample collections

After seven days treatment, blood and liver samples were collected after terminal anaesthesia by intraperitoneal injection of 10% chloral hydrate. Blood was collected into a tube without anticoagulants and then centrifuged at 3500 rpm for 15 min to collect serum for further analysis. Liver samples were weighted, fixed with 10% paraformaldehyde or frozen in −80 °C for histological analysis and protein isolation.

### Serum and liver tissues biochemical indices

ALT, AST, ALP, TBA, TG, TBIL, TP and TC were measured by automatic blood biochemical analyser (HITACHI 7020; Hitachi, Chiyoda, Japan) according to the manufacturer’s protocols.

Liver samples (100 mg) were homogenized in 0.9 mL normal saline, and the mixture was centrifuged at 12,000 rpm for 20 min to collect the supernatant, as described before (Yang et al. 2017). Serum levels of TBA, TG and TC in the liver tissues were determined by automatic biochemical analyser.

### Histopathological examination

The liver samples were fixed with 10% formalin for 72 h and then embedded in paraffin to be sliced at 5 μm using an R2235 rotary microtome (Leica, Wetzlar, Germany). The sections were stained with haematoxylin and eosin (H&E) and examined under a BX51 light microscope with DP71 electronic camera system (Olympus, Tokyo, Japan).

### Western blot assay

Liver tissues (about 20–30 mg) were lysed with RIPA and PMSF, and then placed on the ice for 15 min. Then tissue homogenate was centrifuged at 12,000 rpm for 20 min at 4 °C to collect the supernatant. Proteins were separated by 4–12% SDS polyacrylamide gel electrophoresis and transferred onto polyvinylidene difluoride (PVDF) membranes. The membranes were blocked with 5% (w/v) skimmed milk for 2 h at room temperature and probed overnight at 4 °C with a primary antibody. Then, the membrane was incubated with species-specific secondary antibodies, HRP-conjugated anti-rabbit IgG and HRP-conjugated anti-mouse IgG for 1–2 h at room temperature. The membrane was treated with enhanced chemiluminescence (ECL) and blots were detected by the Amersham Imager 680. The densitometry of the protein blot was analysed by Image J (Bethesda, MD).

### The HPLC–MS/MS method of BAs detection

The concentration of BAs was determined by HPLC–MS/MS (API 4000, SCIEX, Framingham, MA). The liquid chromatography column (100 × 3 mm, 2.5 μm) is from Phenomenex (Torrance, CA). The column temperature was set to 30 °C, the flow rate was set to 0.3 mL/min, and the injection volume was 10 μL. A gradient elution program was optimized and run as listed followed by using a mobile phase consisting of 0.05% HCOOH-H_2_O (A) and 0.05% HCOOH-CH_3_CN (B): 0–0.1 min, 22%B; 0.1–6 min, 25%B; 6–15 min, 30%B; 15–25 min, 35% B; 25–35 min, 50% B; 35–45 min, 95%B; 45–46.0 min, 95% B; 46.0–46.1 min, 22% B; 46.1–50 min, 22% B.

For MS detection, the ionization mode was chosen in negative; the capillary voltage was set at −4500 V; source temperature was 300 °C; scan type was MRM; gas I, gas II, CUR and CAD were 60.0, 75.0, 10.0 and 5.0 psi, respectively.

### Preparation of liver samples and standard stock solutions

Fresh liver samples (15–20 mg) were mixed with 100 ng/mL internal standard (50 μL) and 0.1 M NaOH (250 μL). The samples were incubated in an oven at 80 °C for 1 h and then mixed with 2% acetonitrile solution (700 μL). The mixture was centrifuged at 14,000 rpm for 5 min to collect the supernatant.

Bile acid standard stock solutions were prepared by dissolving the respective 29 BAs and a certain amount of 11 internal standard BAs in acetonitrile. The concentration gradients in the standard mixed solution were 0.1, 1, 10, 20, 50, 100, 500, 1000 and 2000 ng/mL, and the concentration of the standard internal solution was 30 ng/mL.

### Analysis of BSEP and MRP2 transport activity

Each experiment of transporter BSEP, MRP2 or MRP4 was divided into three groups: (1) negative control group (NC): 0.05 mg vesicles and pure blank buffer A2 and 10 μM substrate TCA/E_2_17βG/E_2_17βG for BSEP, MRP2 or MRP4, respectively. (2) Inhibitor group: 0.05 mg vesicles, 10 μM substrate and inhibitor 200 μM rifampin/100 μM benzbromarone/200 μM indomethacin for BSEP, MRP2 or MRP4, respectively. (3) Isopsoralen group: 0.05 mg vesicles, 10 μM substrate and 10 μM isopsoralen. The time of reaction is 5 min.

The vesicles on the filter plate were dissolved in 50 μL 80% methanol and centrifuged at 2000 rpm for 2 min, and repeated this operation once. The methanol solution collected twice was combined and pre-cooled methanol containing internal target was added. The supernatant was centrifuged and the amount of substrate in the sample was detected by HPLC–MS/MS (API 4000, SCIEX, Framingham, MA). The liquid chromatography column (30 mm × 2 mm, 4 μm) is from Phenomenex (Torrance, CA). The mobile phase consisted of water (A) (containing 0.01% ammonia) and methanol (B). The column temperature was set to 40 °C, the flow rate was set to 0.5 mL/min and the injection volume was set to 5 μL. The activity of transporter was assessed by the amount of substrate that was transported into the vesicles. The relative activity was normalized to the transport activity of NC group.

### Data analysis

Statistical analysis was performed using GraphPad Prism software, version 7.0 (La Jolla, CA). Data presented in the form of bar graphs are mean ± standard error of the mean (SEM). Statistical analyses between two groups were performed using Student’s two-tailed *t*-test. Statistics were performed using GraphPad Prism (La Jolla, CA), and *p*< 0.05 was considered statistically significant.

## Results

### Effects of isopsoralen on body weight and liver/body weight ratio

After three-day-administration, the body weight and liver weight had no significant difference compared with those of control group. After seven-day-administration, the body weight and liver weight both decreased significantly. But there was no significant difference in liver/body weight between the three groups (Table 1).

**Table 1.** Effects of isopsoralen on body weight, liver weight and liver/body weight ratio.

	Body weight (g)		Liver weight (g)	Liver/body weight (%)
	Before treatment	After treatment		
Cont	19.44 ± 0.74	16.01 ± 0.62	0.91 ± 0.07	5.67 ± 0.45
3d	19.99 ± 0.76	15.85 ± 1.31	0.90 ± 0.08	5.71 ± 0.92
7d	19.80 ± 1.29	15.00 ± 0.80**	0.81 ± 0.13*	5.42 ± 0.72

Data are represented as mean ± SD (*n* = 14, half female and half male).

*p< 0.05, **p< 0.01, indicate significant difference compared with control group.

### Effects of isopsoralen on serum biochemical indices

**Figure 1.**
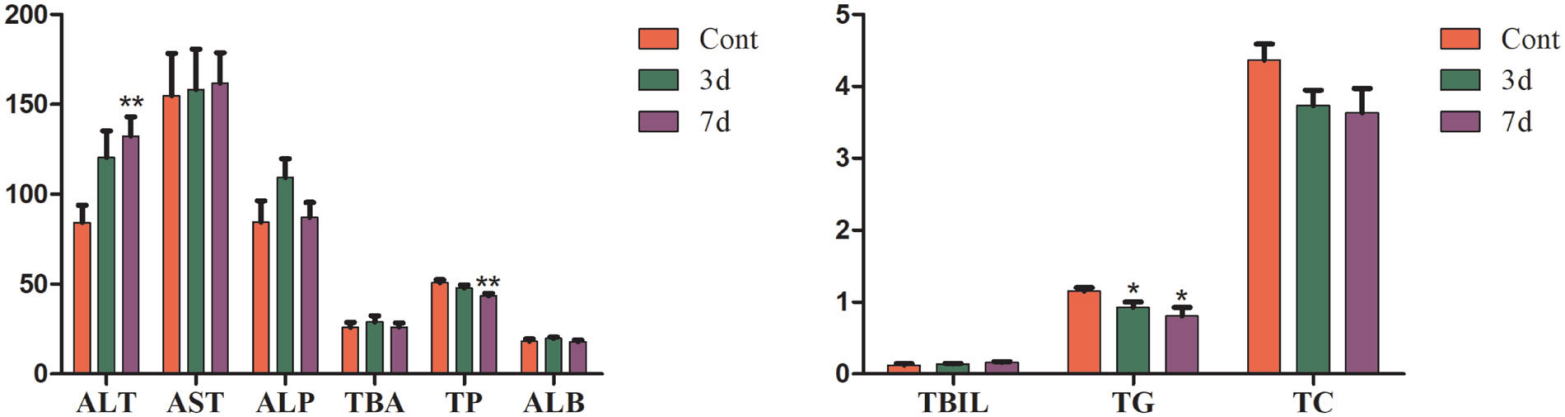
Effects of isopsoralen on serum biochemical indices. ALT (u/L), AST (u/L), ALP (u/L), TBA (mmol/L), TP (u/L), ALB (g/L), TBIL (μmol/L), TG (mmol/L) and TC (mmol/L). Data are shown as mean ± SEM (*n* = 14, half female and half male). *p< 0.05, **p< 0.01 and ***p< 0.001 indicate significant difference from the control group.

As shown in Figure 1, after administration with isopsoralen for three days, the levels of serum ALT increased significantly while the levels of serum ALP, TBA, TP, TC and ALB decreased significantly compared with those of control group. After administration for seven days, the levels of serum ALT and AST increased significantly while the levels of serum TBA, TP and ALB decreased significantly. The serum TBIL and TG had no obvious change.

### Histopathological changes in liver

**Figure 2.**
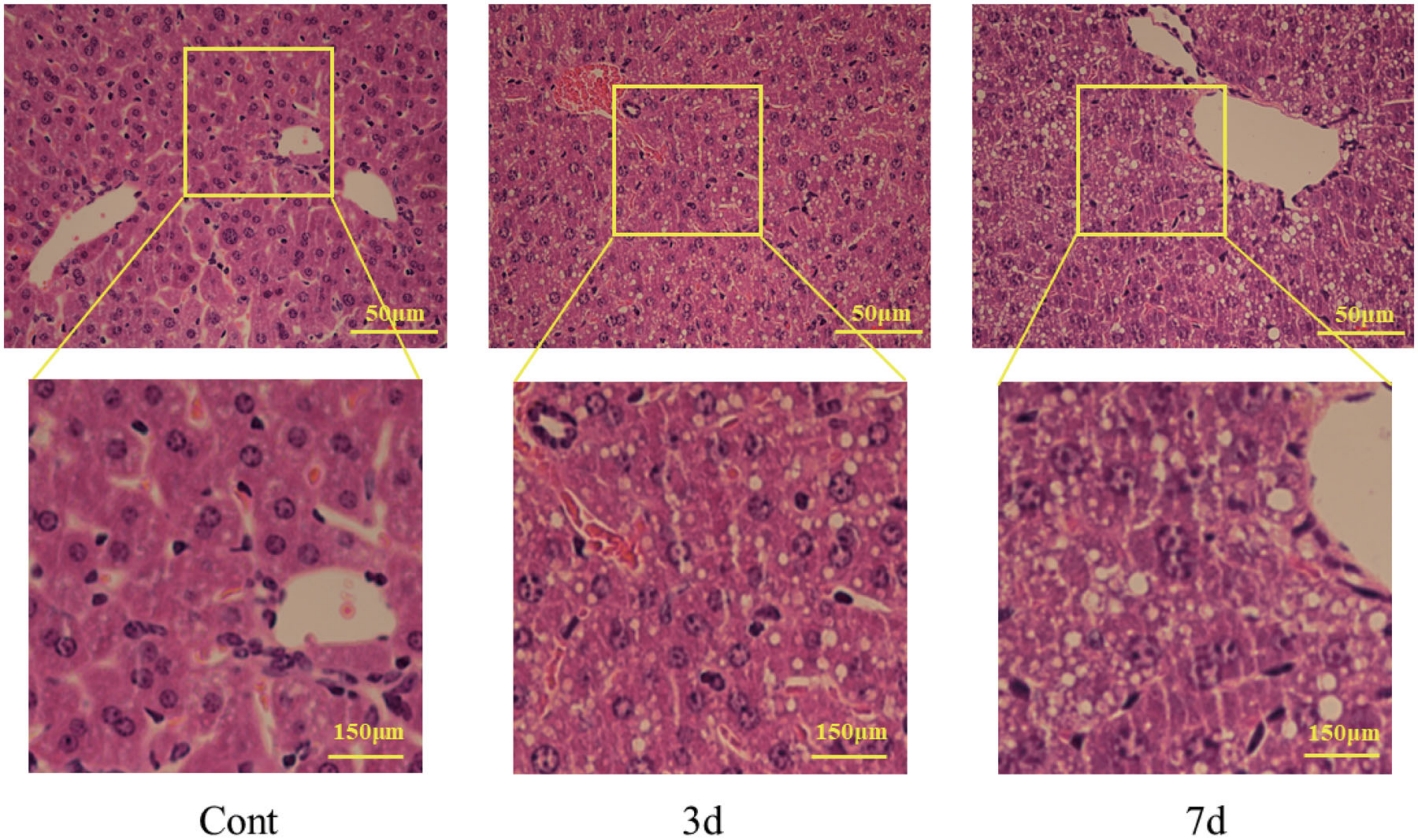
The sections of mice liver before and after isopsoralen-treatment with H&E staining. Cont: control group; 3d: 3d group; 7d: 7d group.

The liver of mice in control group is normal under optical microscope, and the structure around central vein and portal area were normal, and the hepatic lobule structure was clear, and the cells were intact without degeneration. After three-day-administration with isopsoralen, the hepatocytes appeared vacuolar degeneration and swelling obviously. After seven-day-administration, severe vacuolar degeneration of hepatocytes was observed and the area of vacuolar degeneration increased in liver tissue (Figure 2).

### Effects of isopsoralen on lipid contents in liver tissues

**Figure 3.**
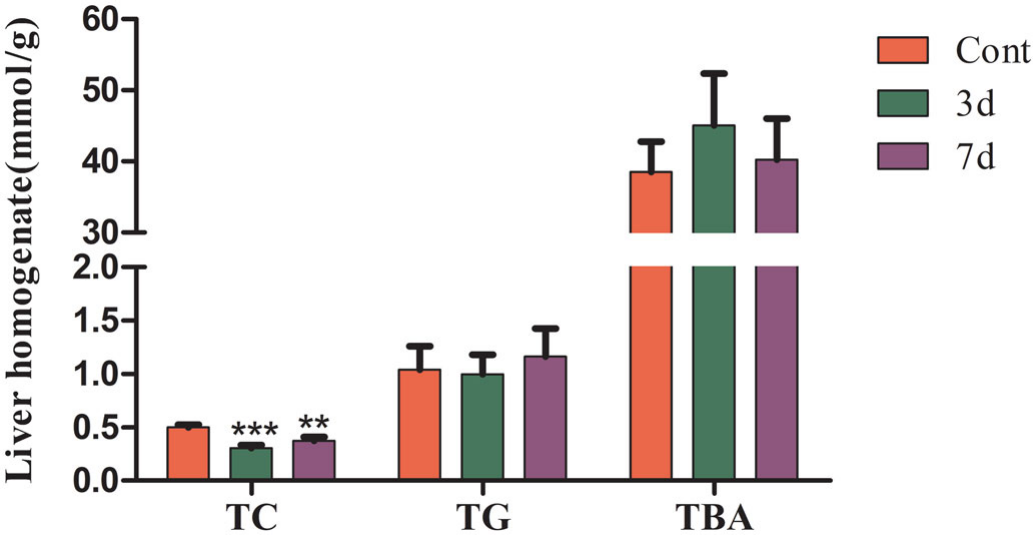
Lipid contents in liver homogenate from mice after isopsoralen administration for 3 days and 7 days. Data are represented as the mean ± SEM (*n* = 14, half female and male mice). **p < 0.01 and ***p < 0.001 compared with control group.

After administration with isopsoralen, the level of hepatic TC of mice significantly decreased in three-day group and seven-day group. There were no significant changes in TG and TBA, although the level of TBA showed an increasing trend (Figure 3).

### Effects of isopsoralen on the protein levels of hepatic bile acid transporters in mice

**Figure 4.**
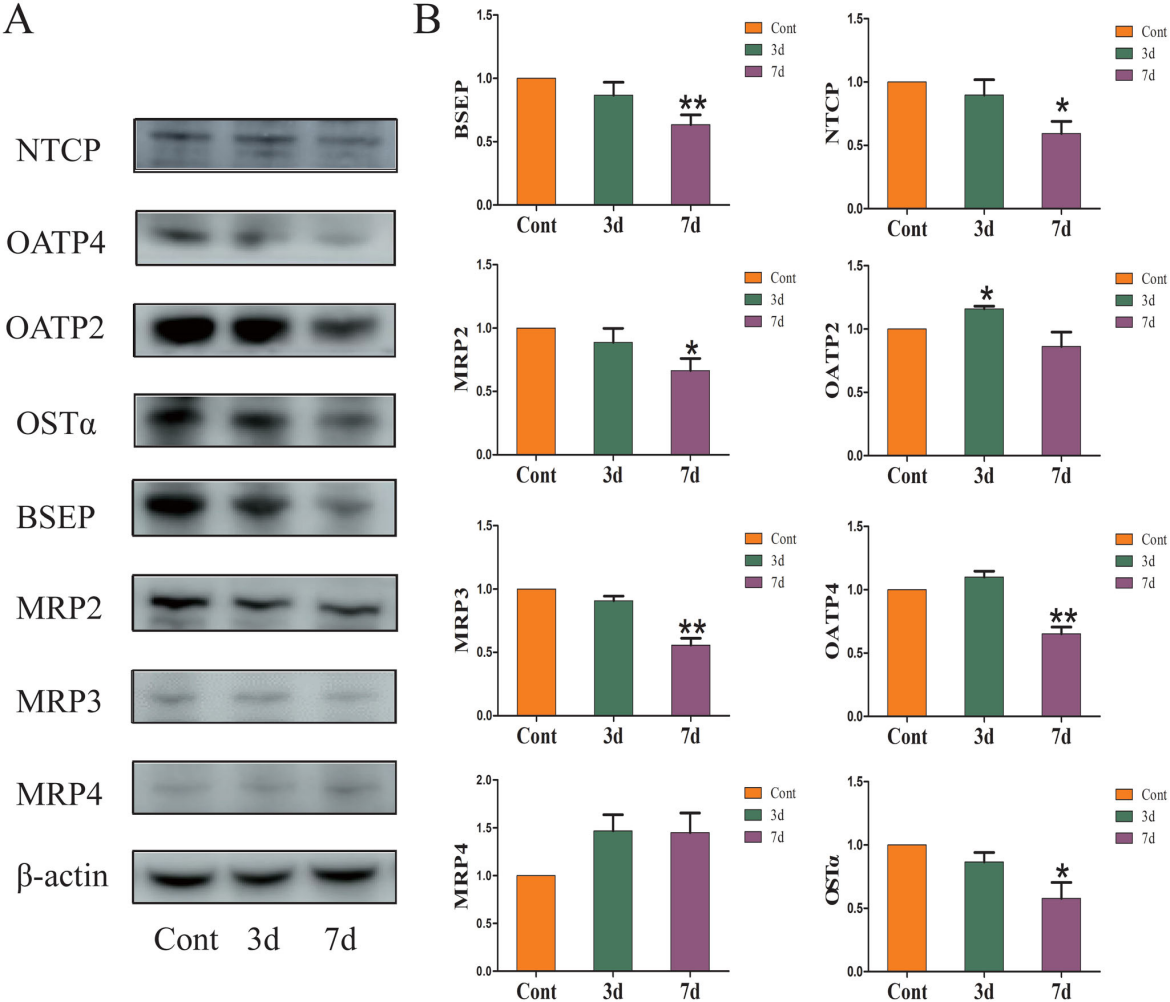
Changes of expression levels of hepatic transporters in the livers. (A) Protein expression levels of hepatic BA transporters as shown by western blot. (B) Relative expression levels of BA transporters to β-actin. The value is expressed as the mean ± SEM, *n* = 6. The mean value of the control group was normalized (all recorded as 1), *p< 0.05, **p< 0.01 indicate significant difference.

The effects of isopsoralen on the BA transporters are shown in Figure 4. After isopsoralen administration for three days, the expression of OATP2 increased significantly, while the expression of NTCP and OATP4 did not change significantly. After isopsoralen treatment for seven days, the expression of BSEP, MRP2, MRP3, OSTα, NTCP and OATP2 was inhibited, although the expression of MRP4 was increased, there was no significant difference.

### Effects of isopsoralen on the protein levels of hepatic bile acid receptor and binding enzyme in mice

**Figure 5.**
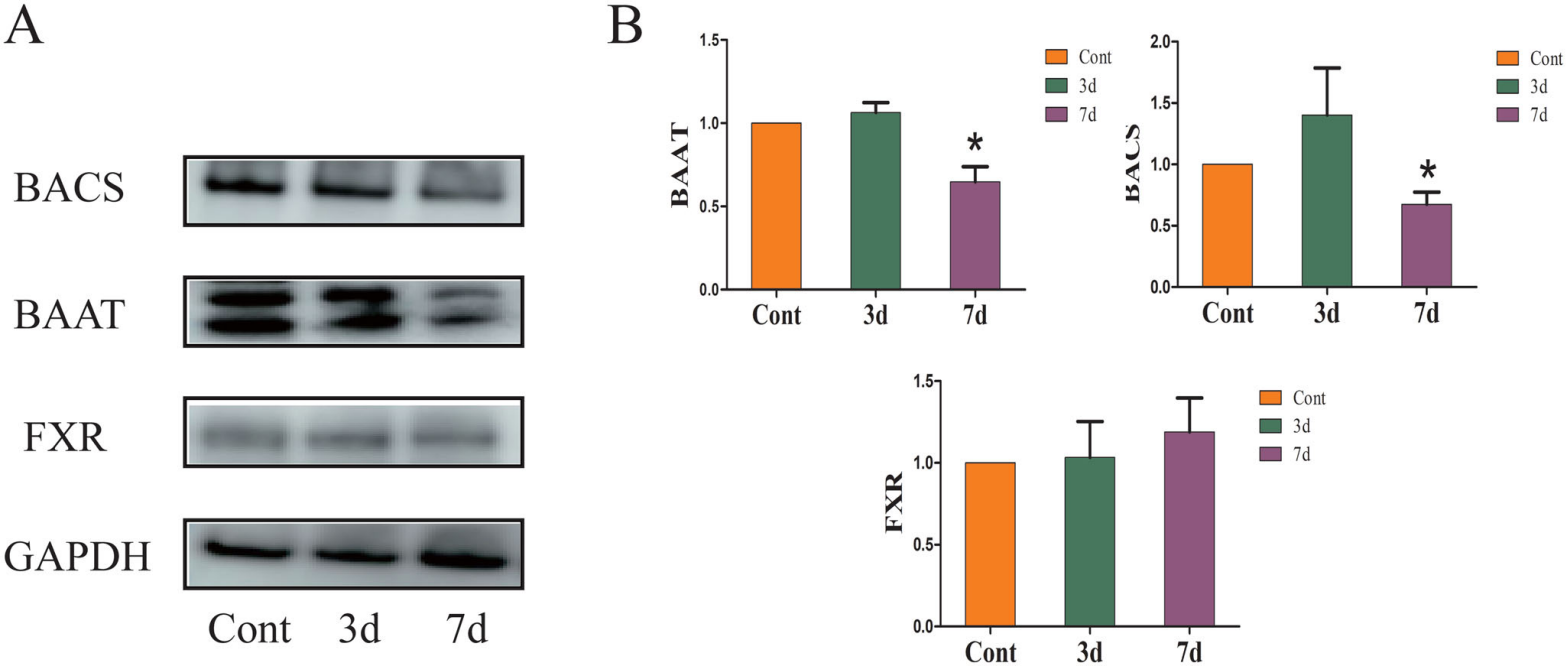
Changes of expression levels of bile acid receptor and binding enzyme in the livers. (A) Protein expression levels as shown by western blot. (B) Relative expression levels of FXR, BAAT and BACS to β-actin. The value is expressed as the mean ± SEM, *n* = 6. The mean value of the control group was normalized (all recorded as 1), *p< 0.05 indicate significant difference.

As shown in Figure 5, after administration of isopsoralen for seven days, the expressions of BAAT and BACS were significantly decreased while there was no significant difference with FXR.

### Changes of bile acid profiles in liver

**Figure 6.**
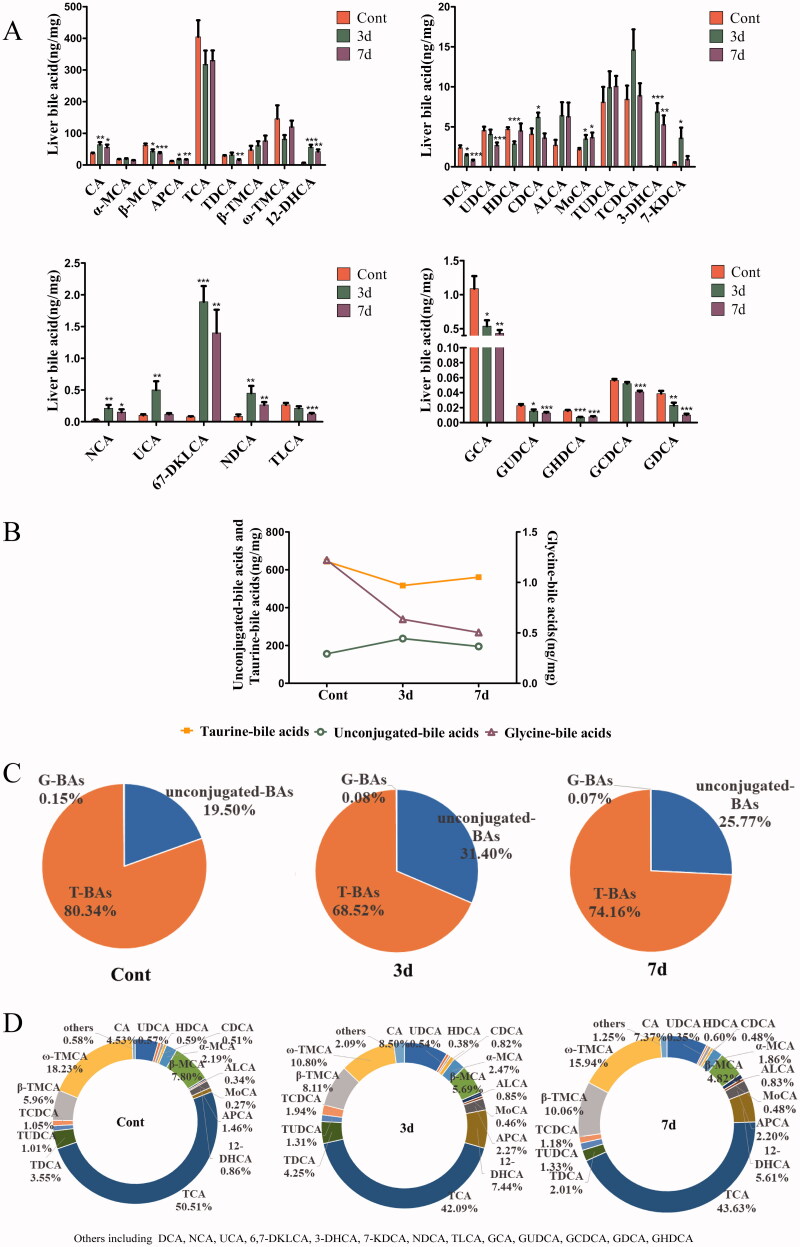
Changes of bile acids concentrations and compositions in mice liver after isopsoralen treatment analysed by UHPLC–MS/MS. (A) Changes of contents of each BA. (B) Changes of total amount of unconjugated-BAs, taurine-BAs and glycine-BAs upon isopsoralen administration. (C) Changes of proportion of unconjugated-BAs, taurine-BAs and glycine-BAs in total BAs before and after isopsoralen administration. (D) The proportions of each BA as percentage of total bile acid before and after isopsoralen administration. Data represented as mean ± SEM (*n* = 14, half female and male mice). *p< 0.05, **p< 0.01 and ***p< 0.001, significantly different compared with control group.

In order to determine whether the effects of isopsoralen on the liver are accompanied by changes in BAs, we qualified 29 BAs in liver by HPLC–MS/MS. The contents of CA, APCA, 12-DHCA, CDCA, MoCA, 3-DHCA, 7-DHCA, NCA, UCA, 6,7-DKLCA and NDCA significantly increased after isopsoralen treatment. By contrast, β-MCA, DCA, UDCA, HDCA, TDCA, TLCA and 5 glycine-BAs significantly decreased. Besides, mice with isopsoralen treatment also partly increased ALCA, TUDCA, TCDCA and T-βMCA, but without statistical significance (Figure 6(A)). After three-day-administration, the total amount of unconjugated-BAs in 29 BAs increased, while the total amount of taurine-BAs and glycine-BAs decreased (Figure 6(B)). We found that taurine-BAs accounted for the major portion of all measured BAs. The total amount of unconjugated-BAs in the treatment group was higher than those in the control group. On the contrary, the conjugated-BAs were lower than those in the control group (Figure 6(C)). After isopsoralen treatment for seven days, the total amount of taurine-BAs decreased from 80.34% to 74.16% and glycine-BAs decreased from 0.15% to 0.07%, while unconjugated-BAs increased from 19.50% to 25.77% (Figure 6(C)). The proportion of CA, MoCA, TUDCA and T-βMCA in the TBAs increased in a time-dependent manner (Figure 6(D)).

### Inhibition of isopsoralen on the transport activities of BSEP, MRP2 and MRP4

**Figure 7.**
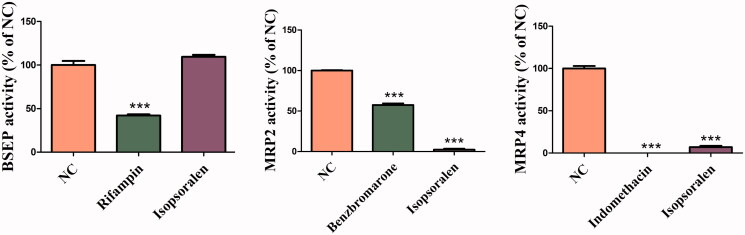
Effects of isopsoralen on the transport activity of bile acid transporters. The values are expressed as the mean ± SEM (*n* = 3). ***p< 0.01 indicates significant difference.

As shown in Figure 7, under the concentration of 10 μM, isopsoralen had obvious inhibitory effect on the transport activity of MRP2 and MRP4, but not on BSEP.

## Discussion

Isopsoralen is one of the main bioactive compounds and quality control compounds of FP. However, with the expanded application of FP, its toxicity has also gradually become the focus of clinicians. Liver, which is the important metabolic organ that eliminates foreign substances, is usually the primary target of drug toxicity and is highly susceptible to drug damage (Piñeiro-Carrero and Piñeiro 2004). Although there has been reported that isopsoralen could affect the expression of mRNA in BA transporters, it was only preliminary investigation, and the change of protein level has not been verified. Moreover, the synthesis and regulation of BAs were not considered. According to the Chinese Pharmacopeia (2020 edition), the content of psoralen and isopsoralen should be not <0.7% in PF. In toxicological safety studies, the choice of the trial dose is usually higher than the clinical equivalent dose. 80 mg/kg isopsoralen is equivalent to about 22.86 g crude drug, which is equivalent to 2.3–2.9 times the clinical dose. Therefore, the dose of 80 mg/kg is worthy of safety evaluation. The present study assesses the toxic effect of isopsoralen (80 mg/kg) in C57BL/6J mice after short-term administration on hepatic transporters and BA profiles.

Although there were no significant changes in body weight, liver weight and organ coefficient in three-day group, body weight and liver weight were significantly reduced after seven-day-administration, suggesting that the liver may be damaged. Besides, histopathological examination revealed obvious vacuolization, swelling and necrosis of liver cells after three days of isopsoralen administration, and the area of necrosis lesions further expansion after seven days of isopsoralen administration. These changed parameters confirmed that isopsoralen has hepatotoxicity, which is consistent with previous research (Wang Y et al. 2019; Zhang Y, Yuan, et al. 2019; Zhang Y, Zhang, et al. 2019). Furthermore, isopsoralen induced deranged liver function after three-day-administration, and this abnormal change had appeared in the early stage of using isopsoralen, which should be paid enough attention.

Liver injury usually also assessed by study of hepatocyte integrity markers (ALT, AST), cholestatic parameters (bilirubin, ALP) and hepatic synthetic function (ALB, TP) (Giannini et al. 1999; Chambers et al. 2011; Penndorf et al. 2013; Okubo et al. 2016). In this study, isopsoralen significantly increased the level of ALT in three-day and seven-day group, while it also rose the level of AST in seven-day group. On the contrary, the levels of TP, TC, TBA and ALB were evidently decreased. ALT is mainly present in cytoplasm while AST is mainly present in mitochondria. When liver cells are mildly damaged, the level of ALT rises earlier than AST (Anderson et al. 2000). Moreover, since TP and ALB are synthesized from liver parenchymal cells, our results indicated that isopsoralen negatively affected liver and the ability of hepatocyte synthesis was impaired. In the liver homogenate, the expression of TC was inhibited but TG had no significant change, which indicated that isopsoralen may affect the synthesis of cholesterol. Although TBA did not increase significantly, we speculated that the accumulation of BA in liver was not obvious due to the administration time being too short.

Liver is in dynamic equilibrium with BAs under normal circumstances, and this process is regulated by different BA transporters. It has been shown previously that the abnormal of bile secretion pathway was associated with isopsoralen-induced liver injury (Zhang Y, Zhang, et al. 2019). When the transport system of BAs is impaired, BA circulation is also disrupted which induced the apoptosis of hepatocyte and damage to the liver (Trauner et al. 1998). The elimination of drugs and metabolites from liver back to the bloodstream is accomplished mainly by MRP3, while the elimination of the biliary canaliculi is mediated by MRP2. Moreover, the BSEP is mainly eliminated BA efficiently (Jetter and Kullak-Ublick 2020). In the experiment of transporter activity, isopsoralen inhibited the transport activities of BSEP, MRP2 and MRP3 significantly, suggesting that isopsoralen could affect the expression of BA transporters *in vivo*.

Similarly, the expression of BSEP and MRP2 was also inhibited significantly after seven-day-administration in C57BL/6J mice, suggesting that isopsoralen could inhibit the excretion of BAs. It is worth noting that the level of MRP3 and MRP4 generally is very low under normal conditions. When cholestasis occurs in the liver, the expression of MRP3 and MRP4 will be stimulated to reduce the accumulation of BAs in the liver (Jetter and Kullak-Ublick 2020). However, isopsoralen also significantly inhibited MRP3 and OSTα after administration in this study. Although the expression of MRP4 was increased, there was no significant difference compared with control group. This may be because the administration time was short and the accumulation of BA in liver was not serious.

Bile acid uptake transporters NTCP, OATP2 and OATP4 are mainly located in the basolateral membrane of hepatocytes, responsible for transporting bile salts, bilirubin and other substances in blood to hepatocytes (Alrefai and Gill 2007). In the research of hepatic cholestasis, the expression of NTCP was usually inhibited (Zollner et al. 2001). The mechanism may be related to the negative feedback regulation of BA receptors in the liver, such as FXR, fibroblast growth factor 5 (FGF5), pregnane X receptor (PXR), etc., which reduces BA uptake by the liver and regulates BA balance. We found that the expression of NTCP and OATP4 was significantly decreased after seven-day-administration, which indicated that the BA content of hepatocytes from hepatic portal vein was decreased. However, the expression of FXR did not change significantly, we speculate that the inhibition of NTCP and OATP4 expression after isopsoralen administration may be due to the regulation of other receptors, this needs further exploration. The abnormal expression of BA transporters suggested that the homeostasis of BAs in the liver may also have changed.

Bile acids, derived from cholesterol, can be used as detergents for the absorption and metabolism of nutrients, fats and vitamins. Also, BAs were used as multifunctional signal molecules to activate specific ligands of nuclear and membrane receptors (Chiang 2009). Unconjugated-BAs usually combine with glycine (G-BAs) or taurine (T-BAs) under the effect of BAAT and BACS in the liver, and most BAs in the body are in the form of conjugated BAs (García-Cañaveras et al. 2012). Hydrophobicity is an important factor in BA hepatotoxicity (Heuman 1989). It may cause hepatocyte necrosis or apoptosis by destroying cell membrane, promoting the production of reactive oxygen species, and causing mitochondrial dysfunction and endoplasmic reticulum stress (Perez and Briz 2009). Some studies have confirmed that the toxicity of bile salts increases with the increase of hydrophobicity (Morita et al. 2019). After three-day-administration, the content of unconjugated-BAs increased in 29 BAs, especially the CA and CDCA which with high toxicity. However, the contents of T-BAs and G-BAs were decreased, this result indicated that isopsoralen may destroy the binding of unconjugated-BAs with taurine or glycine. After seven-day-administration, the significant decrease in the expression of BAAT and BACS may be the direct cause of the increase of unconjugated-BAs. After seven-day-administration, the content of unconjugated-BAs was lower than that at three-day-administration, while the content of taurine-BAs was higher than that at three-day-administration. This change may be related to the significant decrease of NTCP expression and the significant increase of OATP2 at three-day-administration.

When the contents of TCDCA, TDCA, CDCA and other hydrophobic BAs increase, the liver mitochondrial function will be damaged, showing obvious hepatotoxicity (Sagawa et al. 1993; Rolo et al. 2000). In this study, the content of CA, and CDCA increased significantly after isopsoralen administration, and TCDCA tended to increase, suggesting that liver injury may be related to the increase of these hydrophobic BAs. On the other hand, hydrophilic BAs can protect the liver from damage. UDCA is a hydrophilic BA and could regulate hepatocyte injury induced by other hydrophobic BAs and stimulates the expression of BSEP, MRP2 and MRP3 (Fickert et al. 2001; Zollner et al. 2003). In this study, the significant decrease in UDCA expression and the inhibitory expression of BSEP, MRP2 and MRP3 may also be a potential cause of liver damage caused by isopsoralen. Therefore, the accumulation of toxic BAs may be the factor of liver injury caused by isopsoralen.

However, the composition of BAs is associated not only with liver, but also with gut flora. The size of BA pools depends on the microbial community in the gut (Sayin et al. 2013). Secondary BAs, which are hydrolysed by the intestinal flora, are reabsorbed by ASBT and OSTα/β in the ileum and colon back to liver through the portal vein. We can be further speculated that, after intragastric administration of isopsoralen, the activity of intestinal flora, as well as the expression of BA-related transporters in the intestinal tract also changed. Therefore, in order to further understand how isopsoralen changes BA composition and induces liver injury, intestinal BA spectrum and BA transporters under physiological and pathological conditions needs to be compared.

## Conclusions

This study confirmed that 80 mg/kg isopsoralen can induce liver injury in C57BL/6J mice after a short time administration. The hepatotoxicity of isopsoralen is related to the inhibition of BAs-related transporters, resulting in the disruption of BAs spectrum and the accumulation of some hydrophobic BAs in the liver.
